# Epistatic Interplay between Type IV Secretion Effectors Engages the Small GTPase Rab2 in the *Brucella* Intracellular Cycle

**DOI:** 10.1128/mBio.03350-19

**Published:** 2020-03-31

**Authors:** Erin P. Smith, Alexis Cotto-Rosario, Elizabeth Borghesan, Kiara Held, Cheryl N. Miller, Jean Celli

**Affiliations:** a Paul G. Allen School for Global Animal Health, Washington State University, Pullman, Washington, USA; Tufts Medical School; Fred Hutchinson Cancer Research Center

**Keywords:** *Brucella*, Rab2, type IV secretion, epistasis, macrophage, pathogenesis

## Abstract

Intracellular bacterial pathogens remodel cellular functions during their infectious cycle via the coordinated actions of effector molecules delivered through dedicated secretion systems. While the function of many individual effectors is known, how they interact to promote pathogenesis is rarely understood. The zoonotic bacterium Brucella abortus, the causative agent of brucellosis, delivers effector proteins via its VirB type IV secretion system (T4SS) which mediate biogenesis of the endoplasmic reticulum (ER)-derived replicative *Brucella*-containing vacuole (rBCV). Here, we show that T4SS effectors BspB and RicA display epistatic interactions in *Brucella* replication. Defects in rBCV biogenesis and *Brucella* replication caused by deletion of *bspB* were dependent on the host GTPase Rab2a and suppressed by the deletion of *ricA*, indicating a role of Rab2-binding effector RicA in these phenotypic defects. Rab2a requirements for rBCV biogenesis and *Brucella* intracellular replication were abolished upon deletion of both *bspB* and *ricA*, demonstrating that the functional interaction of these effectors engages Rab2-dependent transport in the *Brucella* intracellular cycle. Expression of RicA impaired host secretion and caused Golgi fragmentation. While BspB-mediated changes in ER-to-Golgi transport were independent of RicA and Rab2a, BspB-driven alterations in Golgi vesicular traffic also involved RicA and Rab2a, defining BspB and RicA’s functional interplay at the Golgi interface. Altogether, these findings support a model where RicA modulation of Rab2a functions impairs *Brucella* replication but is compensated by BspB-mediated remodeling of Golgi apparatus-associated vesicular transport, revealing an epistatic interaction between these T4SS effectors.

## Abstract (precis)

Bacterial pathogens with an intracellular lifestyle modulate many host cellular processes to promote their infectious cycle. They do so by delivering effector proteins into host cells via dedicated secretion systems that target specific host functions. While the roles of many individual effectors are known, how their modes of action are coordinated is rarely understood. Here, we show that the zoonotic bacterium Brucella abortus delivers the BspB effector that mitigates the negative effect on bacterial replication that the RicA effector exerts via modulation of the host small GTPase Rab2. These findings provide an example of functional integration between bacterial effectors that promotes proliferation of pathogens.

## INTRODUCTION

Intracellular bacterial pathogens have developed an array of mechanisms to exploit and redirect host cellular functions toward achieving their infectious cycle. For this purpose, many use dedicated secretion systems that deliver effector proteins into host cells and which individually target and modulate specific host functions to promote bacterial survival, persistence, or proliferation. Identification and characterization of individual effectors are instrumental in deciphering the molecular mechanisms used by these pathogens to exploit specific host functions and yet do not encompass how the delivered effectors combine their modes of action to support the bacterium’s intracellular cycle. Instances of cooperative or antagonistic interactions between effectors have highlighted the importance of their combined functions in modulating cellular pathways. These include effectors in Legionella pneumophila that directly or indirectly modulate the activity of other effectors to ensure spatiotemporal regulation of intracellular events (1–6). Epistatic, antagonistic actions between effectors that indirectly compensate each other’s activities on specific intracellular stages have also been characterized in Salmonella enterica and L. pneumophila (7, 8).

The zoonotic bacterium Brucella abortus survives and proliferates within host phagocytes by generating a replication-permissive organelle derived from the host endoplasmic reticulum (ER), the replicative *Brucella*-containing vacuole (rBCV), via remodeling of its original endosomal BCV (eBCV) (9). Conversion of eBCV to rBCV is driven by the *Brucella* VirB type IV secretion system (T4SS) (10–13), which delivers effector proteins (14–19) that are thought to remodel BCV trafficking and promote bacterial replication. rBCV biogenesis and bacterial replication require functions of the host early secretory pathway, including vesicular transport steps controlled by the host small GTPases Sar1, Rab1, Rab2, and Arf1 (20–22). Although little is known about the roles and modes of actions of *Brucella* type IV effectors, recent studies have identified some that may target host secretory functions. These include BspA, BspB, and BspF, which impair host secretory trafficking and contribute to bacterial replication (17), and RicA, which binds Rab2 and downmodulates *Brucella* replication in HeLa cells (14). Among these, BspB alters secretory traffic via its interaction with the Golgi apparatus-associated conserved oligomeric Golgi (COG) complex, a regulator of vesicular traffic at, and within, the Golgi apparatus (23) and causes redirection of COG-dependent vesicular traffic to promote rBCV biogenesis and bacterial replication (22). These findings argue that *Brucella* delivers a series of effector proteins that modulate specific host secretory functions to mediate rBCV biogenesis and possibly bacterial replication within rBCVs. Whether, and how, *Brucella* T4SS effectors coordinate to integrate their respective modes of action toward promoting the bacterium’s intracellular cycle is unknown.

RicA binds the GDP-bound inactive form of Rab2 *in vitro* and promotes Rab2 recruitment to the BCV (14), suggesting that it modulates Rab2-dependent transport as part of its mode of action. How RicA modulates Rab2 functions and whether it interferes with Rab2-dependent secretory transport remain unknown. Interestingly, the replication defect of a Δ*bspB* mutant is restored by depletion of Rab2a in macrophages (22), suggesting a functional link between BspB and Rab2-dependent transport and possibly RicA. We therefore examined the relationship between these two T4SS effectors during *Brucella* infection. Here, we reveal a functional epistatic interplay between these effectors, where BspB and RicA engage Rab2-dependent functions in the *Brucella* intracellular cycle and BspB compensates RicA’s deleterious activity on rBCV biogenesis and *Brucella* replication. These findings uncover how *Brucella* T4SS effectors finely tune their actions to modulate host secretory transport for bacterial replication purposes.

## RESULTS

### BspB is dispensable for rBCV biogenesis and bacterial replication in the absence of RicA.

**FIG 1:**
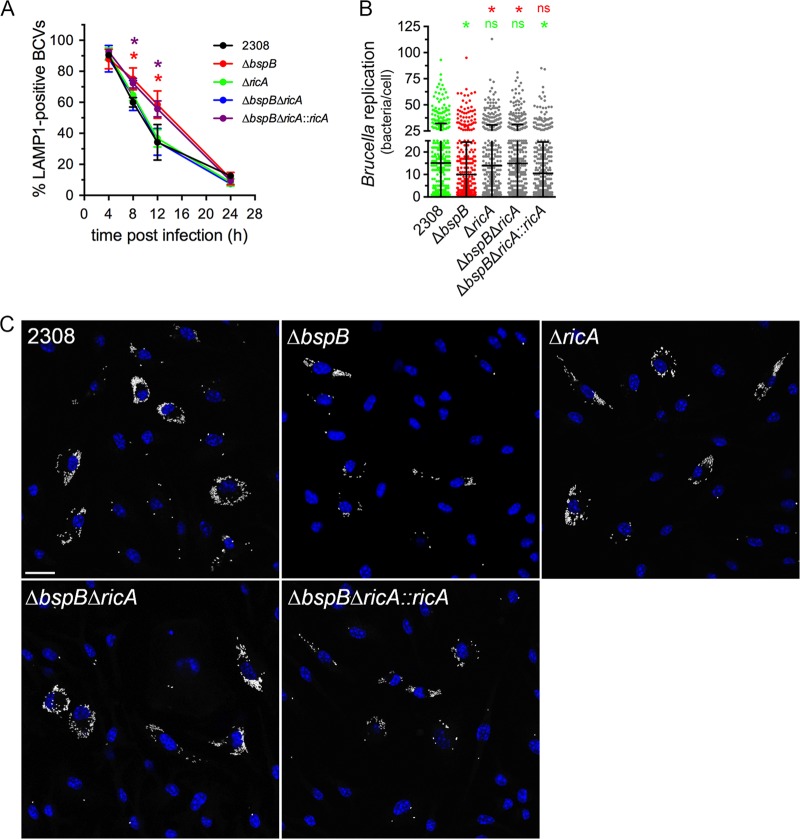
Deletion of *ricA* renders *bspB* dispensable for rBCV biogenesis and replication of B. abortus. (A) rBCV biogenesis in BMMs infected with DsRed_m_-expressing wild-type (strain 2308), Δ*bspB*, Δ*ricA*, Δ*bspB* Δ*ricA*, or Δ*bspB* Δ*ricA*::*ricA* B. abortus strains, as measured by acquisition and then exclusion of LAMP1-positive membranes. Data are means ± SD of results from 3 independent experiments. Asterisks indicate a statistically significant difference between wild-type (2308) and Δ*bspB* (red) or Δ*bspB* Δ*ricA*::*ricA* (purple) bacteria, determined by two-way ANOVA with Dunnett’s multiple-comparison test. *, *P* = <0.05. (B) Intracellular replication of DsRed_m_-expressing wild-type (2308), Δ*bspB*, Δ*ricA*, Δ*bspB* Δ*ricA*, or Δ*bspB* Δ*ricA*::*ricA* B. abortus strains in BMMs at 24 h pi. Values are means ± SD of results from at least 3 independent experiments. Asterisks indicate a statistically significant difference compared to either the wild-type (2308; green) or Δ*bspB* (red) populations, assessed using a nonparametric Kruskal-Wallis test with Dunn’s multicomparison statistical analysis. ns, not significant. (C) Representative confocal fluorescence micrographs of BMMs infected with DsRed_m_-expressing wild-type (2308), Δ*bspB*, Δ*ricA*, Δ*bspB* Δ*ricA*, or Δ*bspB* Δ*ricA*::*ricA* B. abortus strains (pseudocolored in white) for 24 h pi. Nuclei were stained using Hoechst 33342 (blue). Scale bar, 20 μm.

To determine whether BspB and RicA functionally interact, we first used a genetic approach to compare RicA’s role in rBCV biogenesis and bacterial replication to that of BspB in primary murine macrophages. As previously described (22), Δ*bspB* bacteria underwent delayed rBCV biogenesis compared to wild-type bacteria, as visualized by a slower exclusion of LAMP1-positive endosomal membranes from BCVs between 8 and 12 h postinfection (pi) (Fig. 1A) and a replication defect at 24 h pi (Fig. 1B and C). In contrast, Δ*ricA* bacteria displayed rBCV biogenesis kinetics and replication similar to those of the wild-type bacteria (Fig. 1), indicating that RicA is not essential for rBCV biogenesis and intracellular replication of *Brucella* in primary macrophages. Similar replication results were obtained in HeLa cells (see Fig. S1A in the supplemental material), except that BspB was also dispensable for bacterial replication, ruling out this infection model as appropriate to study BspB-RicA interactions. Interestingly, the deletion of *ricA* in Δ*bspB* bacteria suppressed the delayed rBCV biogenesis and bacterial replication defect caused by deletion of *bspB* seen in bone marrow-derived macrophages (BMMs), indicating that BspB is dispensable for these intracellular events in the absence of RicA. This suppressive effect was due to *ricA* deletion, as it was complemented by reintroduction of a chromosomal copy of *ricA* in *trans* (Fig. 1). The suppressive effect of *ricA* deletion was specific to BspB-dependent replication, as it failed to restore normal growth of a replication-deficient strain that lacks BspF (strain Δ*bspF*; Fig. S1B), another T4SS effector interfering with host secretion (17). Hence, these results demonstrate a genetic link between BspB and RicA functions and suggest that BspB may counteract RicA activities that interfere with rBCV biogenesis and *Brucella* replication.

10.1128/mBio.03350-19.1 FIG S1 B. abortus replication and effect of *ricA* deletion in HeLa cells and BMMs. (A) Bacterial replication of B. abortus wild-type, Δ*bspB*, Δ*ricA*, and Δ*bspB* Δ*ricA* strains in HeLa cells. Values represent numbers of bacteria in individual cells at 12 h pi and are from at least 3 independent experiments. Replication levels at 12 h pi in HeLa cells were comparable to those in BMMs at 24 h pi. (B) Bacterial replication of B. abortus wild-type, Δ*bspF*, Δ*bspF*::*bspF*, and Δ*bspF* Δ*ricA* strains in BMMs. Values represent numbers of bacteria in individual cells at 24 h pi and represent results from 3 independent experiments. Download FIG S1, TIF file, 0.2 MB. Copyright © 2020 Smith et al. 2020 Smith et al. This content is distributed under the terms of the Creative Commons Attribution 4.0 International license.

### RicA and BspB engage Rab2-dependent functions in *Brucella* replication.

**FIG 2:**
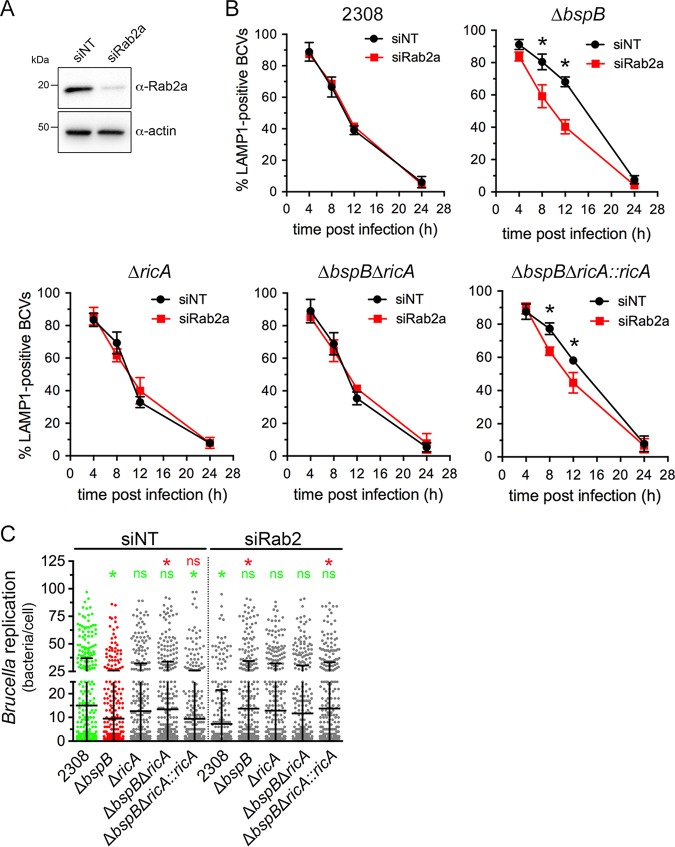
Rab2a depletion renders BspB dispensable for rBCV biogenesis and *Brucella* replication. (A) Representative Western blotting of Rab2a depletion in BMMs. BMMs were nucleofected with either small interfering nontargeting (siNT) or siRab2a siRNAs, and Rab2 levels were evaluated after 72 h via Western blotting of Rab2a and β-actin as a control. (B) rBCV biogenesis in BMMs treated with either nontargeting (siNT) or siRab2a siRNAs for 72 h and infected with DsRed_m_-expressing wild-type (2308), Δ*bspB*, Δ*ricA*, Δ*bspB* Δ*ricA*, or Δ*bspB* Δ*ricA*::*ricA* B. abortus strains. Data are means ± SD of results from 3 independent experiments. Asterisks indicate a statistically significant difference determined by two-way ANOVA with Dunnett’s multiple-comparison test. (C) Intracellular replication of DsRed_m_-expressing wild-type (2308), Δ*bspB*, Δ*ricA*, Δ*bspB* Δ*ricA*, or Δ*bspB* Δ*ricA*::*ricA* B. abortus strains at 24 h pi in BMMs treated with either nontargeting (siNT) or siRab2a siRNAs for 72 h prior to infection. Values are means ± SD of results from at least 3 independent experiments. Asterisks indicate a statistically significant difference compared to siNT-treated BMMs infected with either wild-type (2308; green) or Δ*bspB* (red) strains, assessed using a nonparametric Kruskal-Wallis test with Dunn’s multicomparison statistical analysis; ns, not significant.

Given the role of Rab2 in *Brucella* replication (21, 22) and RicA binding to Rab2 (14) and considering that BspB targets the COG complex, which orchestrates Rab2-dependent Golgi apparatus-ER vesicular transport, we next examined the effect of Rab2-dependent transport inhibition on the intracellular behavior of BspB-deficient and RicA-deficient bacteria in BMMs. In contrast with a previous study performed in HeLa cells showing a role of Rab2 in rBCV biogenesis (21), small interfering RNA (siRNA)-mediated depletion of Rab2a in BMMs (86.0% ± 12.7% depletion; Fig. 2A) did not affect rBCV biogenesis by wild-type B. abortus (Fig. 2B), despite efficiently inhibiting Golgi apparatus-ER retrograde transport (22). However, Rab2 depletion (89.0% ± 2.4%) significantly decreased bacterial replication (Fig. 2C), as previously reported (22). Hence, Rab2a appears to be dispensable for rBCV biogenesis in BMMs but required for *Brucella* replication once rBCVs are formed. The absence of RicA did not affect rBCV biogenesis in either small interfering nontargeting (siNT)-treated or siRab2a-treated BMMs (Fig. 2B) and yet rendered *Brucella* replication Rab2a independent (Fig. 2C), suggesting that RicA involves Rab2a functions in bacterial replication. Interestingly, Rab2a depletion suppressed the BCV maturation defect of Δ*bspB* bacteria and caused this otherwise replication-deficient mutant to grow to wild-type levels by 24 h pi (Fig. 2B and C). Deletion of *ricA* in the Δ*bspB* mutant also suppressed BspB’s requirement for rBCV biogenesis and replication and rendered replication of this strain Rab2a independent (Fig. 2B and C). This phenotype was complemented by the chromosomal reintroduction of *ricA* in the Δ*bspB* Δ*ricA* mutant (Fig. 2B and C). Taken collectively, we interpret these findings as showing that RicA involves Rab2a-dependent functions toward rBCV biogenesis and bacterial replication and that BspB counteracts RicA activities on Rab2a-dependent functions that are otherwise deleterious to proper rBCV biogenesis and subsequent replication.

### RicA alters host secretory traffic and Golgi apparatus morphology.

**FIG 3:**
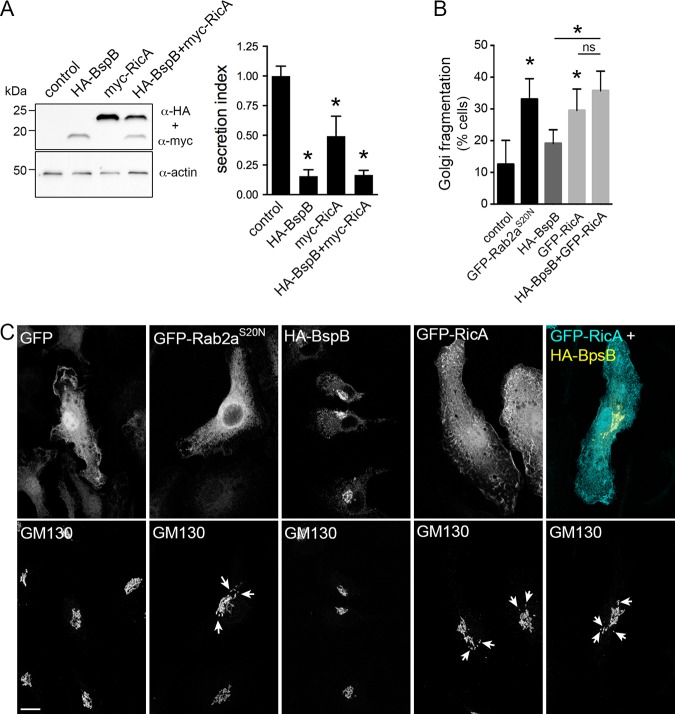
RicA inhibits host secretion and causes fragmentation of the Golgi apparatus. (A) Representative Western blotting of levels of expression of HA-BspB and myc-RicA (individually or in combination) in HeLa cells (left) and analysis of SEAP secretion by HeLa cells expressing HA-BspB and myc-RicA (individually or in combination) (right). Values are means ± SD of results from 3 independent experiments. Asterisks indicate a statistically significant difference compared to control cells, assessed using one-way analysis of variance (ANOVA) followed by a Dunnett’s multicomparison test. (B) Quantification of Golgi fragmentation in BMMs transduced to express GFP (control), GFP-Rab2a^S20N^, HA-BspB, GFP-RicA, or both HA-BspB and GFP-RicA. Fragmentation was scored as presenting at least 4 disconnected GM130-positive structures (30). Values are means ± SD of results from at least 3 independent experiments. Asterisks indicate a statistically significant difference compared to control cells, assessed using one-way analysis of variance (ANOVA) followed by a Dunnett’s multicomparison test; ns, not significant. (C) Representative confocal micrographs of BMMs expressing GFP, GFP-Rab2a^S20N^, HA-BspB, GFP-RicA, or both HA-BspB and GFP-RicA and stained for Golgi apparatus detection using an anti-GM130 antibody. Arrows indicate Golgi fragments. Scale bar, 10 μm.

RicA was identified as binding Rab2 *in vitro* (14), which regulates Golgi apparatus-ER retrograde traffic (24–26), and yet whether RicA activity impairs Rab2-dependent secretory transport has not been established. We first verified the occurrence of RicA-Rab2 interactions in mammalian cells, by coimmunoprecipitation of ectopically expressed myc-tagged RicA with wild-type, dominant-negative (Rab2^S20N^) or constitutively active (Rab2^Q65L^) alleles of green fluorescent protein (GFP)-tagged Rab2 in HeLa cells (Fig. S2), consistent with RicA binding to active or inactive Rab2 *in vivo*. Given that BspB interferes with secretory traffic (17, 22), we then tested the effect of RicA on constitutive host secretion. Expression of myc-tagged RicA in HeLa cells significantly inhibited secretion of a secreted alkaline phosphatase (SEAP) secretory reporter, although not as drastically as expression of hemagglutinin-BspB (HA-BspB) (Fig. 3A) (22), indicating that RicA functionally interferes with the secretory pathway. Coexpression of both myc-RicA and HA-BspB caused SEAP secretion inhibition levels similar to those induced by HA-BspB alone (Fig. 3A), indicating that BspB exerts an inhibitory effect stronger than that exerted by RicA. Since impairment of Rab2 functions causes fragmentation of the Golgi apparatus (27), we next tested the effect of RicA expression of Golgi apparatus morphology. While retroviral expression of HA-BspB in BMMs did not induce any Golgi morphological changes, that of GFP-RicA caused Golgi fragmentation similar to that observed upon expression of dominant-negative GFP-Rab2a^S20N^ (Fig. 3B and C), indicating that RicA expression phenocopies Golgi morphological changes caused by impairment of Rab2a functions. RicA-induced Golgi fragmentation was GFP or cell type independent, as myc-RicA also induced Golgi fragmentation in both BMMs and HeLa cells (Fig. S3). Coexpression of HA-BspB with GFP-RicA in BMMs did not alter the RicA-dependent Golgi fragmentation phenotype (Fig. 3B and C). Taken together, these results show that ectopically expressed RicA interacts with Rab2 and functionally impairs the secretory pathway and yet does so independently of BspB.

10.1128/mBio.03350-19.2 FIG S2 Interaction of RicA with Rab2a alleles in HeLa cells by coimmunoprecipitation. Representative Western blot analyses of coimmunoprecipitations of wild-type (GFP-Rab2a), dominant-negative (GFP-Rab2a^S20N^), or constitutively active (GFP-Rab2a^Q65L^) alleles of Rab2a with myc-RicA were performed. HeLa cells were transfected with the various Rab2a alleles and either myc-RicA or an empty vector control (vector) and subjected to immunoprecipitation of myc-RicA. The respective levels of immunoprecipitated GFP-Rab2a alleles are shown and were quantified by normalization to their expression levels (input) and to the amounts of immunoprecipitated myc-RicA. Download FIG S2, TIF file, 0.2 MB. Copyright © 2020 Smith et al. 2020 Smith et al. This content is distributed under the terms of the Creative Commons Attribution 4.0 International license.

10.1128/mBio.03350-19.3 FIG S3 Similar Golgi fragmentation abilities of myc-RicA and GFP-RicA in HeLa cells and BMMs. Data represent results of quantification of Golgi fragmentation in either transfected HeLa cells (A) or transduced BMMs (B) expressing myc-RicA, GFP, GFP-Rab2a^S20N^ or GFP-RicA, or no protein (vector control). Fragmentation was scored as presenting at least 4 disconnected GM130-positive structures (30). Values are means ± SD of results from at least 3 independent experiments. Asterisks indicate a statistically significant difference between tested conditions, assessed using one-way analysis of variance (ANOVA) followed by a Dunnett’s multicomparison test; ns, not significant. (C) Representative confocal micrographs of HeLa cells expressing myc, myc-RicA, GFP, GFP-RicA, or GFP-Rab2a^S20N^ and stained using an anti-myc antibody and, for Golgi apparatus detection, an anti-GM130 antibody. Scale bar, 10 μm. Download FIG S3, TIF file, 1.4 MB. Copyright © 2020 Smith et al. 2020 Smith et al. This content is distributed under the terms of the Creative Commons Attribution 4.0 International license.

### BspB-mediated alterations of ER-to-Golgi transport are RicA and Rab2a independent.

**FIG 4:**
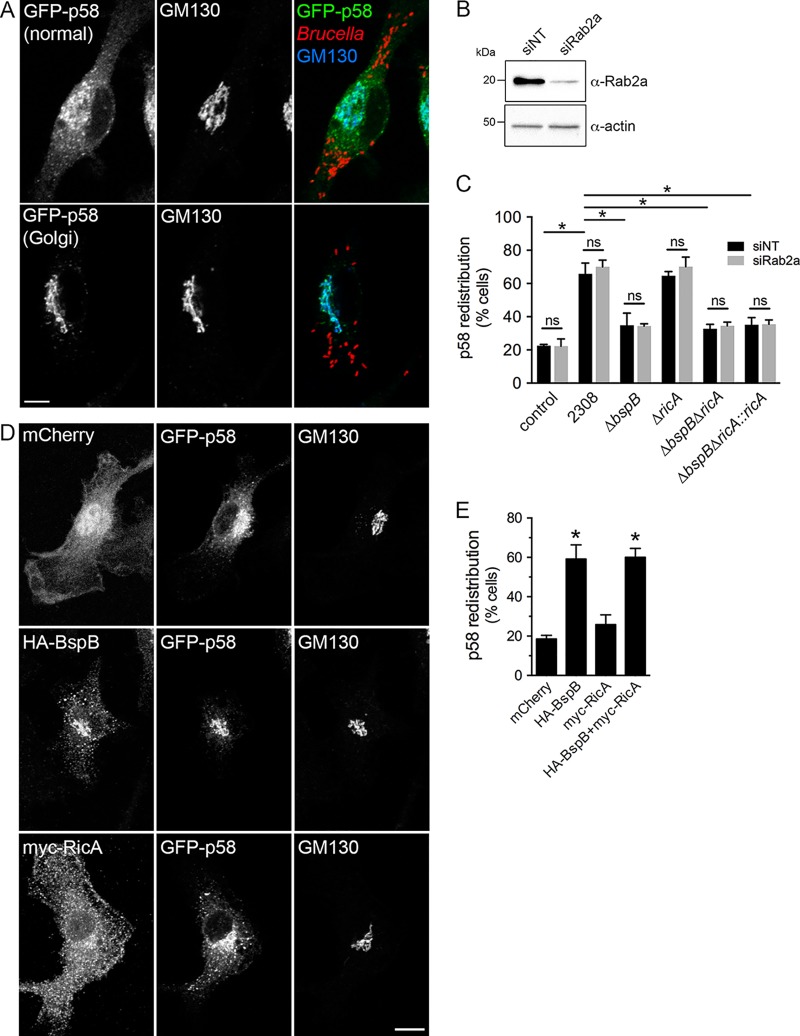
BspB-mediated alterations of ER-to-Golgi transport are independent of RicA and Rab2a. (A) Representative confocal micrographs of GFP-p58 steady-state distribution in *Brucella-*infected BMMs transduced to express GFP-p58, showing either normal or Golgi apparatus-localized patterns. Golgi structures were labeled using an anti-GM130 antibody. Scale bar, 10 μm. (B) Representative Western blotting of Rab2a depletion in BMMs. BMMs were nucleofected with either nontargeting (siNT) or siRab2a siRNAs, and Rab2 levels were evaluated after 72 h via Western blotting of Rab2a and β-actin as a control. (C) Quantification of GFP-p58 redistribution to the Golgi apparatus in BMMs treated with either nontargeting (siNT) or siRab2a siRNAs and then left uninfected (control) or infected for 24 h with wild-type (2308), Δ*bspB*, Δ*ricA*, Δ*bspB* Δ*ricA*, or Δ*bspB* Δ*ricA*::*ricA* B. abortus strains. Values are means ± SD of results from 3 independent experiments. Asterisks indicate a statistically significant difference between tested conditions, assessed using one-way analysis of variance (ANOVA) followed by a Dunnett’s multicomparison test. ns, not significant. (D) Representative confocal micrographs of BMMs cotransduced to express GFP-p58 with mCherry, HA-BspB, or myc-RicA, showing normal or Golgi apparatus-localized distribution of GFP-p58. Scale bar, 10 μm. (E) Quantification of GFP-p58 redistribution to the Golgi apparatus in BMMs cotransduced to express GFP-p58 with mCherry, HA-BspB, myc-RicA, or HA-BspB and myc-RicA. Values are means ± SD of results from 3 independent experiments. Asterisks indicate a statistically significant difference compared to control (mCherry) cells, assessed using one-way analysis of variance (ANOVA) followed by a Dunnett’s multicomparison test. ns, not significant.

To gain a better understanding of the BspB-RicA functional interactions that we uncovered genetically (Fig. 1 and 2), we examined the role of RicA in BspB-dependent changes in secretory transport. BspB causes a redistribution of the ER-to-Golgi intermediate compartment (ERGIC) cargo receptor p58/ERGIC53 to the Golgi apparatus, as a result of altered ER-Golgi secretory transport during infection of BMMs (22). We therefore examined the effect of *ricA* deletion and Rab2a depletion on BspB-mediated p58 redistribution in BMMs. As previously shown (22), *Brucella* infection of BMMs induced p58 redistribution to the Golgi apparatus in a BspB-dependent manner (Fig. 4A and C). Rab2a depletion (93.2% ± 3.0% depletion; Fig. 4B) did not impact these changes (Fig. 4C), indicating that BspB-mediated redistribution of p58 is Rab2a independent. Deletion of *ricA* in either wild-type or Δ*bspB* bacteria did not affect p58 distribution either, compared to the respective parental strains (Fig. 4C). Consistently, retroviral expression in BMMs of HA-BspB, but not of myc-RicA, caused p58 redistribution similar to that seen during infection (Fig. 4D and E). Altogether, these results demonstrate that BspB-mediated changes in ER-Golgi transport are RicA and Rab2 independent and are likely unrelated to BspB-RicA genetic interplay.

### BspB and RicA modulate Golgi apparatus-associated vesicular traffic in a Rab2a-dependent manner.

**FIG 5:**
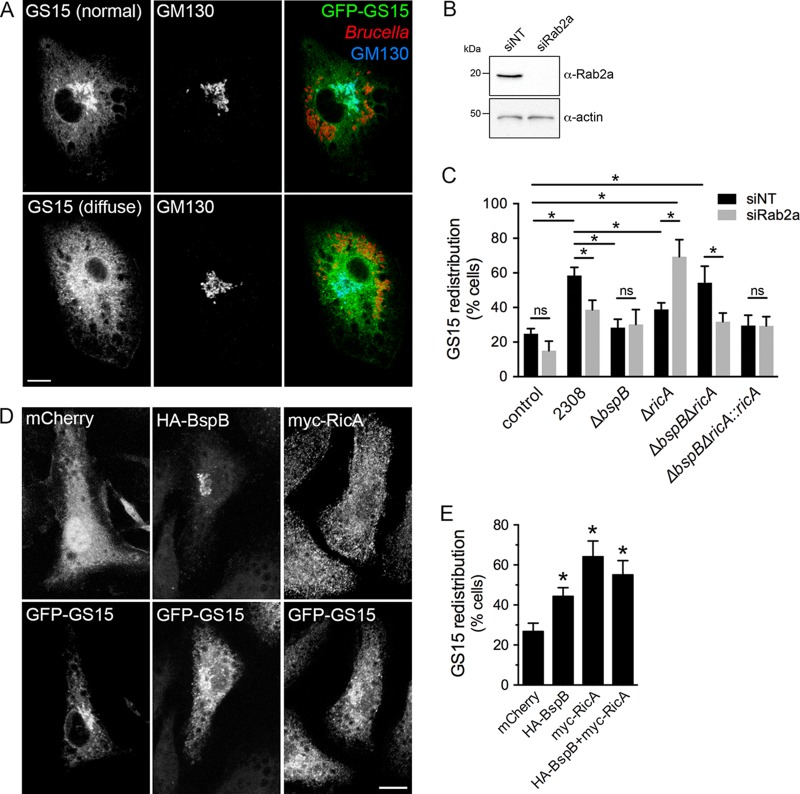
BspB and RicA modulate Golgi apparatus-associated vesicular traffic in a Rab2a-dependent manner. (A) Representative confocal micrographs of GS15 steady-state distribution in *Brucella-*infected BMMs transduced to express GFP-GS15, showing either Golgi apparatus-localized (normal) or diffuse patterns. Golgi structures were labeled using an anti-GM130 antibody. Scale bar, 10 μm. (B) Representative Western blotting of Rab2a depletion in BMMs. BMMs were nucleofected with either nontargeting (siNT) or siRab2a siRNAs, and Rab2 levels were evaluated after 72 h via Western blotting of Rab2a and β-actin as a control. (C) Quantification of GFP-GS15 redistribution from the Golgi apparatus in BMMs treated with either nontargeting (siNT) or siRab2a siRNAs and then left uninfected (control) or infected for 24 h with wild-type (2308), Δ*bspB*, Δ*ricA*, Δ*bspB* Δ*ricA*, or Δ*bspB* Δ*ricA*::*ricA* B. abortus strains. Values are means ± SD of results from 3 independent experiments. Asterisks indicate a statistically significant difference between tested conditions, assessed using one-way analysis of variance (ANOVA) followed by a Dunnett’s multicomparison test. ns, not significant. (D) Representative confocal micrographs of BMMs cotransduced to express GFP-GS15 with mCherry (control), HA-BspB, or myc-RicA, showing normal or diffuse distributions of GFP-GS15. Scale bar, 10 μm. (E) Quantification of GS15 redistribution in BMMs cotransduced to express GFP-GS15 with mCherry, HA-BspB, myc-RicA, or HA-BspB and myc-RicA. Values are means ± SD of results from 3 independent experiments. Asterisks indicate a statistically significant difference compared to control cells, assessed using one-way analysis of variance (ANOVA) followed by a Dunnett’s multicomparison test. ns, not significant.

In addition to its effect on ER-Golgi apparatus traffic, BspB remodels COG-dependent, Golgi apparatus-derived vesicular transport during *Brucella* infection, as measured by the intracellular redistribution of the Golgi SNARE GS15 in BMMs (22). We therefore tested whether RicA and Rab2a play a role in this process, by examining the redistribution of the Golgi SNARE GS15 in control or Rab2a-depleted BMMs. Infection with wild-type bacteria caused a redistribution of GFP-GS15 in BMMs, as previously shown (22), which was partially dependent on Rab2a since its depletion (99.4% depletion; Fig. 5B) decreased GS15 redistribution induced by wild-type bacteria (Fig. 5A to C). Additionally, both Δ*bspB* bacteria and Δ*ricA* bacteria failed to cause GS15 redistribution (Fig. 5A and C) in control (siNT) BMMs, and these defects were genetically complemented in infections with Δ*bspB*::*bspB* and Δ*ricA*::*ricA* bacteria (Fig. S4). This indicates that both BspB and RicA activities contribute to remodeling of Golgi apparatus-derived vesicular traffic. Consistently, the individual or combined forms of retroviral expression of HA-BspB and myc-RicA in BMMs were sufficient to cause GS15 redistribution, confirming the effect of these effectors on Golgi apparatus-associated vesicular traffic (Fig. 5D and E). Interestingly, Rab2a depletion did not impact GS15 redistribution by the Δ*bspB* mutant and yet fully suppressed the redistribution defect caused by the Δ*ricA* mutation, indicating that the activity of RicA, but not that of BspB, in Golgi apparatus-derived vesicular traffic is Rab2a dependent. Furthermore, Δ*bspB* Δ*ricA* bacteria caused GFP-GS15 redistribution similar to that seen with wild-type bacteria (Fig. 5C), indicating that these mutations exert suppressive effects on each other, as seen with rBCV biogenesis and bacterial replication (Fig. 1 and 2). Taken together, these findings indicate that RicA and BspB comodulate Golgi apparatus-derived vesicular transport in a compensatory manner and that RicA, but not BspB, engages Rab2a functions in this process.

10.1128/mBio.03350-19.4 FIG S4 Genetic complementation of the phenotypic defects of B. abortus Δ*bspB* and Δ*ricA* mutants in GS15 redistribution in BMMs. Data represent results of quantification of GFP-GS15 redistribution from the Golgi apparatus in BMMs treated with either nontargeting (siNT) or siRab2a siRNAs and then left uninfected (control) or infected for 24 h with wild-type (2308), Δ*bspB*, Δ*bspB*::*bspB*, Δ*ricA*, or Δ*ricA*::*ricA* B. abortus strains. Values are means ± SD of results from 3 independent experiments. Asterisks indicate a statistically significant difference between tested conditions, assessed using one-way analysis of variance (ANOVA) followed by a Dunnett’s multicomparison test. Values of control, wild-type, Δ*bspB*, and Δ*ricA* strain samples are the same as those included in Fig. 5C and originated from the same sets of experiments. Download FIG S4, TIF file, 0.2 MB. Copyright © 2020 Smith et al. 2020 Smith et al. This content is distributed under the terms of the Creative Commons Attribution 4.0 International license.

## DISCUSSION

Although the role of the *Brucella* VirB T4SS in rBCV biogenesis and bacterial replication has been long known (10–13), only a few VirB effectors dedicated to these processes have been identified (14, 17, 18, 22) and even fewer have been characterized (22), limiting our understanding of the bacterium’s molecular strategies of intracellular proliferation. Here, we show that BspB and RicA, two effectors previously associated with rBCV biogenesis and bacterial replication (14, 17, 22), comodulate Golgi apparatus-associated vesicular transport and engage Rab2-dependent functions in *Brucella* intracellular replication. These findings highlight another level of complexity beyond the individual functions of effectors, whereby the integration of their modes of action finely tunes how the bacterium modulates specific cellular processes.

The concept of interplay between bacterial effectors has been proposed in the context of spatiotemporal regulation of their functions. Several “meta-effectors” have been characterized in L. pneumophila and Salmonella enterica which either regulate the activity of another effector (1, 2, 6) or exert their effect on the activity of another effector via compensatory modification of the same host target (3–5) or via compensatory activity in the same cellular processes (7, 8, 28). Our results argue that the functional interaction between BspB and RicA fits the scenario of compensatory effects of effectors on the same cellular processes. We previously reported that deletion of *bspB* impairs rBCV biogenesis and optimal bacterial replication in macrophages (22), suggesting a direct involvement of BspB in these stages of the bacterium’s infectious cycle. And yet the additional deletion of *ricA* suppresses these phenotypic defects, indicating that BspB’s role in these processes strictly depends upon RicA function, which consequently appears deleterious to rBCV biogenesis and bacterial replication in the absence of BspB. Deletion of *ricA* alone did not confer rBCV biogenesis or replication defects under our experimental conditions, which we interpret as BspB masking negative effects of RicA on rBCV biogenesis and bacterial replication and therefore exerting an epistatic effect on RicA activity. This model of epistatic interplay is supported by the restoration of normal rBCV biogenesis and bacterial replication upon deletion of both *ricA* and *bspB*, which is reminiscent of the genetic and functional interactions between the type III secretion effectors SifA and SseJ in Salmonella enterica and the T4SS effectors SdhA and PlaA in Legionella pneumophila. In these instances, SifA and SdhA promote bacterium-containing vacuole integrity by compensating for the vacuole-disrupting activities of SseJ and PlaA, respectively, and inactivation mediated by the combined effects of both partners in each pair of effectors restores vacuolar integrity (7, 8). In this context, we propose that the combined activities of RicA and BspB contribute to optimal rBCV biogenesis and replication of *Brucella*.

**FIG 6:**
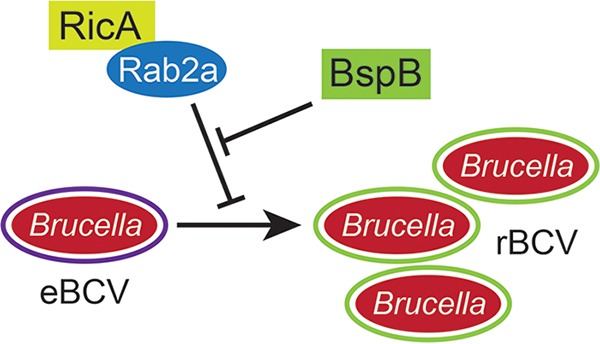
Model of RicA-BspB functional interplay in rBCV biogenesis and *Brucella* replication. RicA modulation of Rab2 functions negatively impacts eBCV-to-rBCV conversion and bacterial replication, which is compensated by BspB activity in Golgi apparatus-associated vesicular transport.

A role for Rab2 in both rBCV biogenesis and bacterial replication was originally uncovered in HeLa cells (21), and we reported its requirement in macrophages previously (22) and in this study but only for optimal bacterial replication of wild-type bacteria. This discrepancy was unlikely to have resulted from an incomplete inactivation of Rab2a functions under our experimental conditions, as we previously showed that siRNA-mediated depletion of Rab2a inhibited Rab2-dependent secretory transport (22). The discovery that RicA is a Rab2-binding effector that negatively modulates rBCV biogenesis and *Brucella* intracellular growth in HeLa cells (14) supports a model whereby RicA negatively affects Rab2 functions that are otherwise required for proper rBCV biogenesis and bacterial replication. Under this assumption, inactivation of Rab2 should phenocopy RicA function. We found instead that Rab2a depletion mimicked RicA deficiency in suppressing rBCV biogenesis and replication defects caused by BspB inactivation, arguing that RicA positively engages Rab2-dependent functions and that BspB activity counteracts RicA’s effect on Rab2-mediated transport to promote rBCV biogenesis and bacterial replication. These findings are consistent with a model in which BspB’s function in rBCV biogenesis and bacterial replication depends upon RicA’s effects on Rab2a-dependent transport. We therefore propose that BspB-RicA functional interplay consists of BspB-mediated compensation for RicA-induced activation of Rab2 functions (Fig. 6). This model predicts that Rab2-dependent functions are dispensable for rBCV biogenesis and bacterial replication. In agreement, we found that Rab2 depletion does not affect rBCV biogenesis by wild-type or RicA-deficient bacteria, only suppressing defects caused by BspB inactivation. However, inactivation of Rab2a functions reduced replication of wild-type bacteria (this study and reference 21) but not that of RicA- or RicA/BspB-deficient bacteria, indicating a requirement for Rab2a functions in replication only in the presence of RicA. This is consistent with effects of RicA activity on Rab2a functions being deleterious to replication in the presence of BspB but not in its absence, further arguing for a combined role of these two effectors during bacterial replication. Alternatively, Rab2a depletion or inactivation may cause indirect effects on secretory transport that are deleterious to *Brucella* intracellular growth, so further studies are required to test this hypothesis.

Our previous studies revealed that BspB alters ER-to-Golgi transport, affecting steady-state localization of ERGIC cargo receptor p58, and also redirects COG-dependent Golgi apparatus-derived vesicular traffic (22). While RicA and Rab2a were not involved in BspB-mediated changes in ER-to-Golgi transport, both contributed to changes in Golgi apparatus-associated traffic, with RicA’s requirement abolished by Rab2a depletion. Similarly to their epistatic interaction in rBCV biogenesis and bacterial replication, *ricA* deletion suppressed the phenotypic defects in GS15 redistribution caused by *bspB* deletion, consistent with counteracting effects of these effectors on this trafficking pathway. These findings highlight this specific stage in secretory transport as a target of BspB and RicA, but whether this common targeting accounts for the functional interplay between these two effectors in rBCV biogenesis and bacterial replication is unknown. The molecular basis of the BspB-RicA functional interaction remains to be understood. BspB interacts with, and alters functions of, the COG complex (22), which coordinates Rab2-dependent vesicular transport from the Golgi apparatus, functionally linking its host target to a Rab2-dependent process. The mode of action explaining the effect of RicA on Rab2 remains elusive. While it is required for Rab2 recruitment on BCVs in HeLa cells (14), whether and how it modulates Rab2 activity are unknown. While a previous study observed a binding preference for GDP-bound, inactive Rab2 *in vitro* (14), our functional data suggest a positive effect of RicA on Rab2 function. Additional work is required to determine whether RicA inhibits or activates Rab2 function during infection. Defining how BspB modulation of COG functions antagonizes RicA’s effect on Rab2 will reveal the molecular basis of their functional interaction. Altogether, our findings illustrate how the functional interactions of T4SS effectors finely tune the modulation of cellular pathways required for the intracellular cycle of *Brucella*.

## MATERIALS AND METHODS

### *Brucella* strains.

Brucella abortus strains 2308, 2308 Δ*bspB*, 2308 Δ*bspB*::*bspB*, and 2308 Δ*bspF* have been described previously (17, 20). To make an in-frame deletion of *ricA*, 1,000-bp regions of the *Brucella* genome upstream and downstream of *ricA* (BAB1_1279) were amplified using primers pairs WSU0345/WSU0346 and WSU0347/WSU0348, respectively. The two resulting fragments were joined by overlap-extension PCR and inserted into pJC80 (20) using XbaI and SacI restriction sites and sequenced to confirm *ricA* in-frame deletion. Strains 2308 Δ*ricA*, 2308 Δ*bspB* Δ*ricA*, and 2308 Δ*bspF* Δ*ricA* were constructed by electroporation of pJC80 Δ*ricA* into B. abortus 2308, B. abortus 2308 Δ*bspB*, and B. abortus 2308 Δ*bspF* strains using SacB-assisted allelic-replacement-based selection as described previously (20). Deletion was confirmed by PCR using primers WSU0345 and WSU0348. *Brucella* strains were modified to express DsRed_m_ through chromosomal insertion of miniTn7K-*dsRed* at the *attTn7* locus through electroporation of *Brucella* strains with pUC18T-miniTn*7*K-dsRed (29) and the corresponding helper plasmid pUC18T-Tn*7*-*tnp*, as described previously (17). For genetic complementation of B. abortus 2308 Δ*bspB* Δ*ricA*, *ricA* preceded by a 489-bp promoter region was amplified from B. abortus genomic DNA using primers WSU0391 and WSU0410 and cloned into pUC18T-miniTn*7*K*-dsRed* using HindIII and KpnI restriction sites. For genetic complementation of B. abortus 2308 Δ*bspF*, *bspF* preceded by a 340-bp promoter region was amplified from pUC18T-miniTn7K-bspF (17) using primers WSU0218 and WSU0219 (Table 1) and cloned into pUC18T-miniTn7K-*dsRed* using EcoRI and KpnI restriction sites. The resulting pUC18T-miniTn*7*K-*dsRed-ricA* and pUC18T-miniTn*7*K-*dsRed-bspF* were electroporated into *Brucella* as described above. All *Brucella* strains were grown on tryptic soy agar (TSA) (BD Difco) for 3 days at 37°C and 5% CO_2_ and in tryptic soy broth (TSB) (BD Difco) at 37°C with shaking until cultures reached an optical density at 600 nm (OD_600_) of ∼1.0 for BMM infections. All experiments with B. abortus strains were performed in a biosafety level 3 facility following CDC Division of Select Agents and Toxins regulations and in compliance with standard operating procedures approved by the Washington State University Institutional Biosafety Committee. For cloning, Escherichia coli strain DH5α (Invitrogen) was grown in Luria-Bertani broth or on Luria-Bertani agar (BD Difco) at 37°C and supplemented with either 50 μg/ml kanamycin or 100 μg/ml ampicillin (Fisher BioReagents) when required.

**TABLE 1:** List of oligonucleotides used in this study

Name	Description	Sequence
WSU0218	*bspF* promoter Forward EcoRI	5′-AGCTCGAATTCACCATCTTCCGATCTTGGCTG-3′
WSU0219	*bspF* Reverse KpnI	5′-AAGGTACCTTATTTATGCTCGGTGAAACTGC-3′
WSU0255	Rab2a Reverse BamHI	5′-CGGGATCCCGTTGGGCAGCTAGACAG-3′
WSU0256	Rab2a SDM S20N Forward	5′-TCGGCGACACAGGTGTTGGTAAAAATTGCTTAT TGCTACAGTTTAC-3′
WSU0257	Rab2a SDM S20N Reverse	5′-GTAAACTGTAGCAATAAGCAATTTTTACCAACA CCTGTGTCGCCGA-3′
WSU0305	Rab2a Forward XhoI	5′-GGCCTCGAGCCATGGCGTACGCCTATCTC-3′
WSU0344	pmCherry N1 Reverse SalI	5′-AGAGTCGACCCGCTACTTGTACAGCTCG-3′
WSU0345	*ricA* del 5′ Forward XbaI	5′-GGCTCTAGACTGCATGGGGTTACGC-3′
WSU0346	*ricA* del 5′ Reverse	5′-CGGCATATGATTTCTCCC-3′
WSU0347	*ricA* del 3′ Forward	5′-GGGAGAAATCATATGCCGTGAAAGGCGGCTGAA-3′
WSU0348	*ricA* del 3′ Reverse SacI	5′-CAGGAGCTCCATCACCGTGAATGC-3′
WSU0349	pmCherry Forward EcoRI	5′-GTCTGAATTCGGTCGCCACCATGG-3′
WSU0353	eGFP C1 Reverse	5′-TGATCAGTTATCTAGATCCGGTGG-3′
WSU0354	eGFP C1 Forward ClaI	5′-TAATATCGATGCCACCATGGTG-3′
WSU0389	*ricA* Forward SalI	5′-CTGGTCGACTATGCCGATCTATG-3′
WSU0391	*ricA* promoter Forward HindIII	5′-CGAAAGCTTAGGAGAATCCGGTTG-3′
WSU0410	*ricA* Reverse KpnI	5′-ATTGGTACCCTTTCAGGCAGGC-3′
WSU0422	pCMV Reverse	5′-CTGCATTCTAGTTGTGGTTTGTCC-3′
WSU0432	*ricA* Forward EcoRI	5′-TCTGAATTCTATGCCGATCTATGC-3′

### Vector construction.

Plasmids pCMV-HA-*bspB*, pEGFP-C1-Rab2a, pEGFP-C1-Rab2a^Q65L^, pCLXSN-HA-*bspB*, pCLXSN-GFP, pCLXSN-GFP-p58, and pCLXSN-GFP-GS15 have been described previously (22). The pCMV-myc-*ricA* plasmid was constructed through PCR amplification of *ricA* from pUC18T-miniTn7K-*dsRed*-*ricA* using primers WSU0389 and WSU0410, and the resulting fragment was cloned into pCMV-myc (Clontech) using SalI and KpnI restriction sites. *ricA* was cloned into pEGFP-C1 (Clontech) using primers WSU0432 and WSU0422 via PCR amplification of *ricA* from pCMV-myc-*ricA*, and the resulting fragment was inserted into pEGFP-C1 using EcoRI and KpnI restriction sites to produce pEGFP-C1-*ricA*. pCLXSN-GFP-*ricA* was constructed via amplification of *eGFP-ricA* from pEGFP-C1-*ricA* using primers WSU0354 and WSU0353 and cloned into pCLXSN-MCS2 (22) using ClaI and BamHI restriction sites. Plasmid pCLXSN-GFP-C1-Rab2a^S20N^ was constructed using site-directed mutagenesis of pCLXSN-GFP-C1-Rab2a (22) (mutation of base pairs 58 to 60 [TCA>AAT]) using primers WSU0256 and WSU0257. The resulting *rab2a^S20N^* cDNA was amplified using primers WSU0305 and WSU0255 and cloned into the pEGFP-C1 vector using BamHI and XhoI restriction sites to make pEGFP-C1-Rab2a^S20N^. The pCLXSN-mCherry plasmid was constructed through amplification of *mCherry* from pmCherry-N1 (Clontech) using primers WSU0349 and WSU0344 and cloned into pCLXSN-MCS2 using EcoRI and SalI restriction sites. All constructs were confirmed by sequencing.

### Mammalian cell culture.

HeLa cells (clone CCL-2) and human embryonic kidney 293T cells (HEK293T/17 clone CRL-11268) were obtained from ATCC and cultured as described previously (22). Primary murine bone marrow-derived macrophages (BMMs) were generated from 6-to-12-week-old female C57BL/6J mice (The Jackson Laboratory) or C57BL/6NHsd mice (Envigo), as described previously (22), following procedures approved by the Institutional Animal Care and Use Committee (IACUC).

### Infection.

BMMs were seeded 2 days prior to infection at 6 × 10^4^ cells/well (24-well plate) and infected with B. abortus strains at a multiplicity of infection (MOI) of 10. Infections were performed as described previously (22). To quantify bacterial replication, coverslips were processed for immunofluorescence microscopy and the number of intracellular bacteria was scored in a blind manner in ∼100 infected cells/experiment within random fields. For rBCV biogenesis, ∼100 intracellular bacteria/experiment were scored in a blind manner for localization within LAMP1-positive BCVs, as previously described (22). Each experiment was repeated independently at least 3 times.

### siRNA treatment and retroviral transduction of BMMs.

Rab2a depletion in BMMs was carried out using a Mouse Macrophage Nucleofector kit (Lonza) and Amaxa Nucleofector with On-Target*plus* SMARTpool siRNAs (Dharmacon) directed against mouse and human Rab2a (L-040851-01-0005 and L-010533-00-0005) or small interfering nontargeting (siNT; J-001810) siRNA, as described previously (22). Knockdown efficiency was evaluated via densitometry in Western blotting using Bio-Rad ImageLab version 4.1 software on a Chemi-Doc gel imaging system (Bio-Rad), normalizing Rab2a expression to that of β-actin for each sample.

Retroviral supernatants were generated in HEK 293T cells transfected for 48 h h with pCLXSN derivatives and the ecotropic helper plasmid pCL-Eco (Retromax, Imgenex), as described previously (22). Retroviral supernatants were added to BMMs (2:5 [vol/vol] ratio) for 48 to 60 h prior to paraformaldehyde (PFA) fixation and 24 to 36 h prior to infection.

### Immunofluorescence microscopy.

Mammalian cells were plated on 12-mm-diameter glass coverslips in 24-well plates at 6 × 10^4^ cells/well for BMMs or 3.5 × 10^4^ cells/well for HeLa cells. Cells were washed 3 times with 1× phosphate-buffered saline (PBS) and then fixed for either 10 min (transductions or transfections) or 20 min (infections) in 3% paraformaldehyde (EMD Millipore) at 37°C. Immunofluorescence staining was performed as described previously (22). Samples were processed in a blind manner and viewed using a Leica DM4000 epifluorescence microscope for quantification and a Leica SP8 confocal laser-scanning microscope for image acquisition. Representative confocal micrographs were acquired at 1,024-by-1,024 pixels and assembled using Adobe Photoshop CS6.

### SEAP secretion assay.

HeLa cells seeded at 3.5 × 10^4^ cells/well (∼50% confluence) in a 24-well plate were cotransfected 24 h after plating with the pSEAP2-Control vector (Clontech; 200 ng) and pCMV plasmid derivatives expressing either *bspB* or *ricA* (300 ng) using FuGENE 6 following the manufacturer’s protocol. SEAP secretion was measured as described previously (17). HA-BspB and myc-RicA expression levels were verified via Western blotting.

### Immunoprecipitation.

HeLa cells were seeded in a 10-cm-diameter tissue culture dish (1 × 10^6^ cells/dish) 24 h prior to transfection. Cells were transfected at ∼70% to 80% confluence following the protocol of the manufacturer of FuGENE 6 and incubated for 18 h at 37°C and 5% CO_2_. Cells were washed 3 times in ice-cold 1× PBS and cross-linked in 1× PBS supplemented with 500 μM dithiobis(succinimidyl-propionate) (DSP) (Thermo Scientific) at 4°C for 2 h. DSP was replaced with ice-cold 20 mM Tris-HCl (pH 7.5), and the cells were further incubated for 15 min at 4°C. Cells were then washed 2 times in ice-cold 1× PBS, lysed in 400 μl of lysis buffer (20 mM Tris-HCl [pH 7.5], 150 mM NaCl, 2 mM MgCl_2_, 0.5% [vol/vol] Triton X-100, 1:500 Halt protease inhibitor [Thermo Scientific]), and incubated at 4°C for 30 min with rotation. Anti-HA-conjugated Dynabeads (Novex, Life Technologies) were blocked in 2% bovine serum albumin (BSA; MP Biomedicals) for 1 h at 4°C with rotation and rinsed 3 times with 1× PBS and once with lysis buffer. Lysates were clarified at 12,000 × *g* for 5 min at 4°C, incubated with beads for 2 h at 4°C with rotation, and then washed 4 times with lysis buffer. Immunoprecipitated proteins were eluted, separated by SDS-PAGE, and analyzed by Western blotting, as described previously (22). Three independent repeats were used to quantify the relative amounts of bound proteins by densitometry using ImageLab version 4.1 software (Bio-Rad). The efficiency of binding of Rab2a alleles to RicA was calculated as the ratio of bound Rab2a/bound RicA after normalization to their respective inputs.

### Antibodies.

The primary antibodies used for immunofluorescence microscopy were rat monoclonal anti-HA (3F10) (1:500; Roche; catalog no. 11867423001), rat monoclonal anti-LAMP1 (1:500; clone 1D4B, obtained from the Developmental Studies Hybridoma Bank, developed under the auspices of the NICHD, and maintained by the University of Iowa Department of Biological Sciences, Iowa City, IA), mouse monoclonal anti-GM130 (1:100; BD Biosciences; catalog no. 610823), and rabbit polyclonal anti-Giantin (1:500; BioLegend; catalog no. 924302). The following Molecular Probes fluorophore-conjugated secondary antibodies were used at 1:500 for immunofluorescence microscopy: Alexa Fluor 488-conjugated donkey anti-rat IgG (catalog no. A21208), Alexa Fluor 594-conjugated donkey anti-rat IgG (catalog no. A21209), Alexa Fluor 488-conjugated donkey anti-mouse IgG (catalog no. A21202), Alexa Fluor 568-conjugated donkey anti-mouse IgG (catalog no. A10037), Alexa Fluor 568-conjugated donkey anti-rabbit IgG (catalog no. A10042), and Cy5-conjugated donkey anti-rabbit IgG (Jackson ImmunoResearch Laboratories; catalog no. 711-175-152).

Primary antibodies for Western blotting were mouse monoclonal anti-myc (9E10) (1:10,000; Thermo Scientific; catalog no. MA1-980), rabbit polyclonal anti-GFP (1:10,000; Molecular Probes; catalog no. A11122), rabbit polyclonal anti-HA (1:10,000; Cell Signaling Technology; catalog no. 3724), mouse monoclonal anti-HA.11 (16B12) (1:10,000; BioLegend; catalog no. 901501), rabbit polyclonal anti-Rab2a (1:1,000; Proteintech; catalog no. 15420-1-AP), and rabbit polyclonal anti-β-actin (1:20,000; Bethyl Laboratories, Inc.; catalog no. A300-485A). The following horseradish peroxidase (HRP)-conjugated secondary antibodies were used at 1:10,000 dilution: horse anti-mouse IgG (Cell Signaling Technology; catalog no. 7076S), goat anti-rabbit IgG (Cell Signaling Technology; catalog no. 7074S), (light-chain-specific) mouse anti-rabbit IgG (Jackson ImmunoResearch Laboratories, Inc.; catalog no. 211-032-171), and (Fcγ fragment-specific) goat anti-mouse IgG (Jackson ImmunoResearch Laboratories, Inc.; catalog no. 115-035-071).

### Quantification and statistical analysis.

Statistical analyses were performed using GraphPad Prism 8 software. Results represent means ± standard deviations (SD) of data from at least three independent experiments. Statistical significance of results of comparisons between treatment groups was determined using one-way analysis of variance (ANOVA) followed by Dunnett’s multiple-comparison test. The specific tests used are indicated in the corresponding figure legends. For bacterial replication, a nonparametric Kruskal-Wallis test with Dunn’s multicomparison statistical analysis was performed, as the values did not follow a normal distribution. For all measurements, a *P* value of <0.05 was considered significant.
