# Supplementary figures and images for: Epistatic Interplay between Type IV Secretion Effectors Engages the Small GTPase Rab2 in the Brucella Intracellular Cycle
Source: mBio. 2020 Mar 31;11(2):e03350-19. doi: 10.1128/mBio.03350-19 (PMC7157780; doi:10.1128/mBio.03350-19)

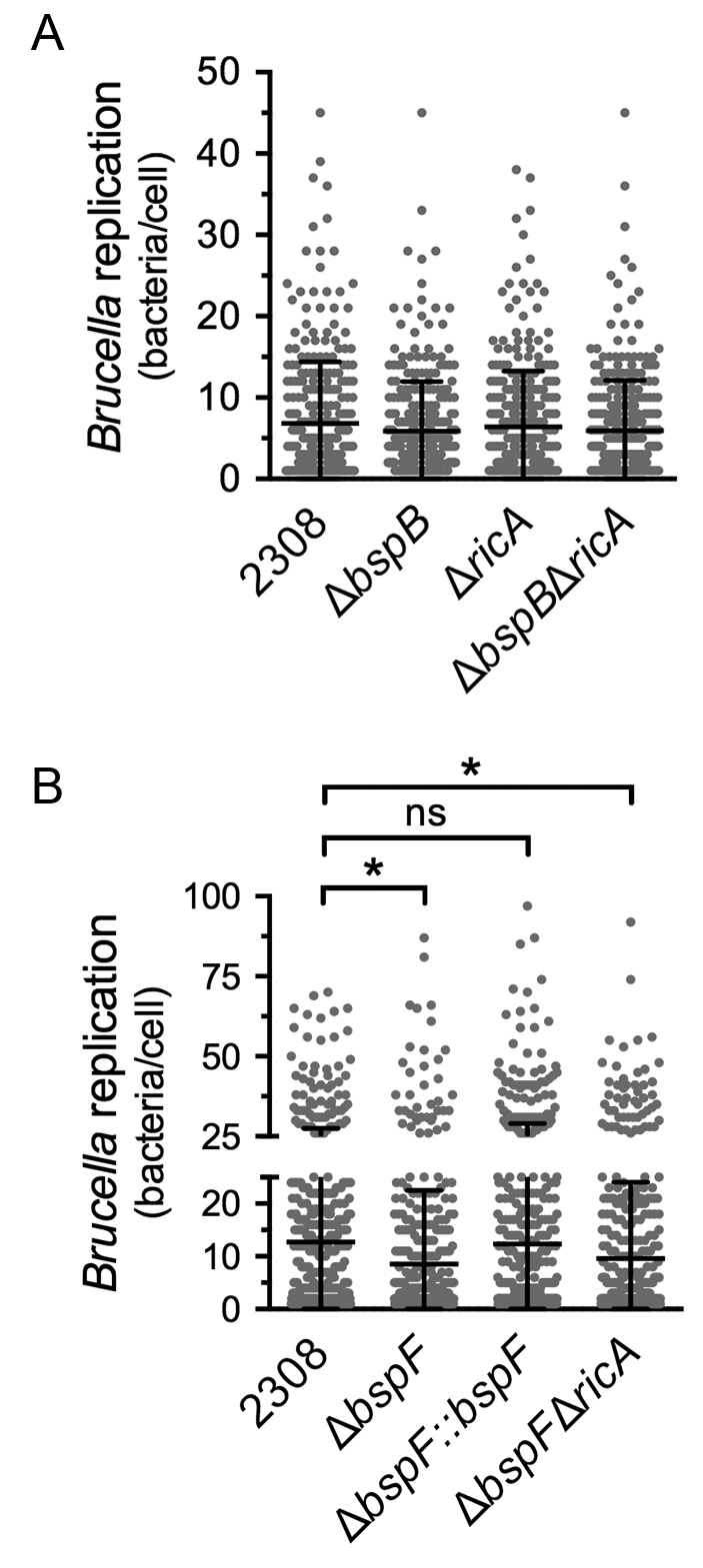

Supplement: FIG S1 [file mBio.03350-19-sf001.tif]

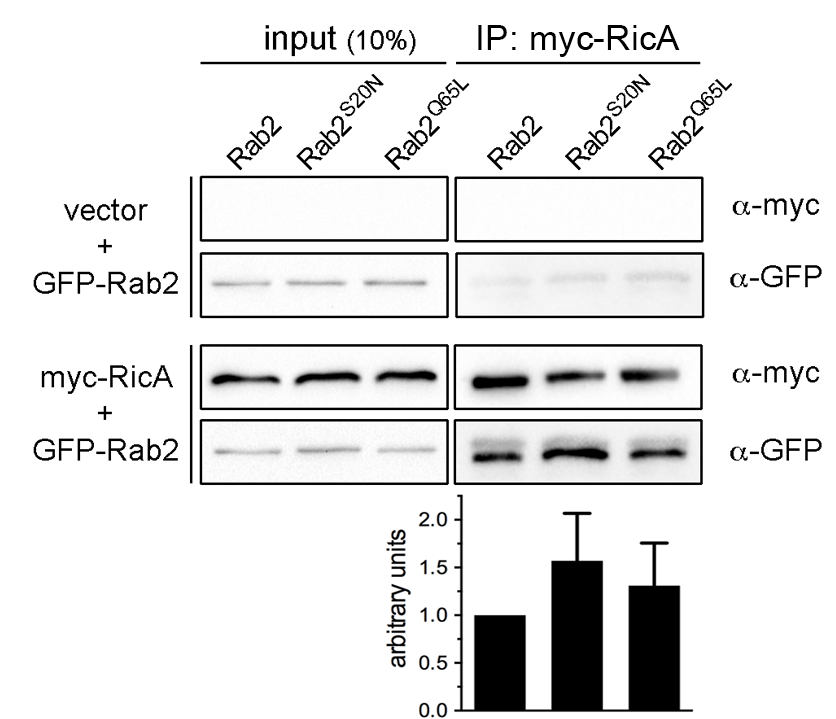

Supplement: FIG S2 [file mBio.03350-19-sf002.tif]

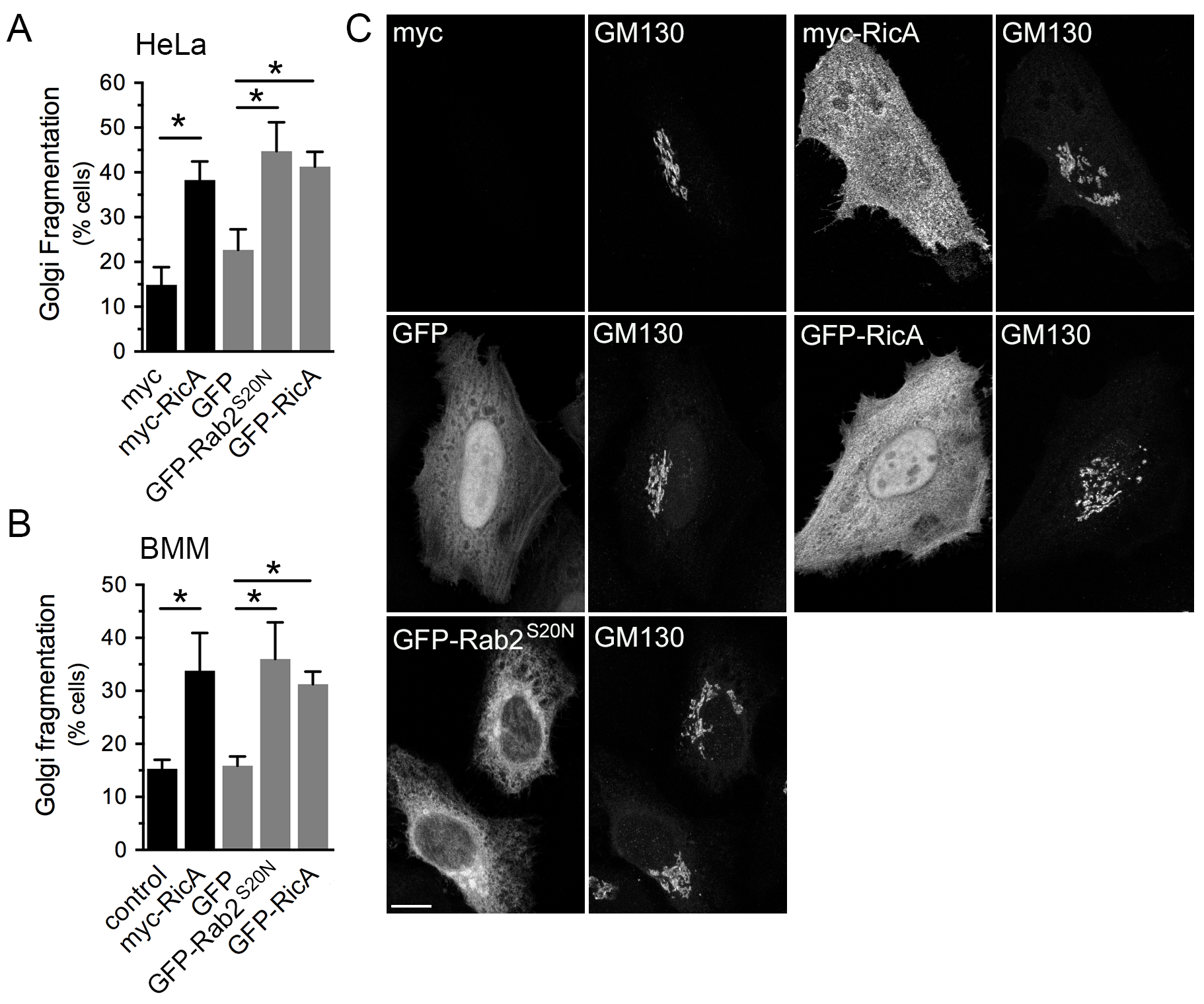

Supplement: FIG S3 [file mBio.03350-19-sf003.tif]

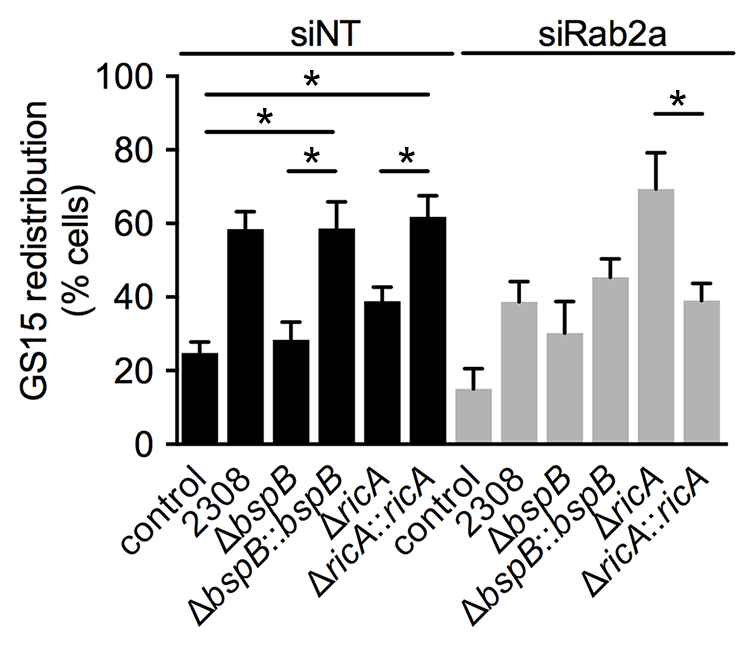

Supplement: FIG S4 [file mBio.03350-19-sf004.tif]
